# American Medical Profession

**Published:** 1855-01

**Authors:** 


### EDITORIAL DEPARTMENT.

### THE AMERICAN MEDICAL PROFESSION.

The members of the American Medical Profession, as a body, present an array of talent, learning and dignified bearing, entitling them to a conspicuous rank in the estimation of the profession throughout the world, and rendering its members worthy the high office they aim to fill and adorn. As the efficient laborers in a profession in its comparative infancy, they well deserve all that is accorded to them when we consider the briefness of its history, the circumstances surrounding it from its earliest date to the present time, the number and value of the contributions it has made, and the improvements effected by it. We avow ourselves partial to our own medical men, and sufficiently "native" to advocate our own claims and defend our national rights, our own Profession always—"*right or wrong*"—and trust that we shall never so far prove recreant to its standing and position as to forget the distinguished efforts of those who have been instrumental in exalting it to the prominent position which it has attained in this country and elsewhere.

The traveler may go abroad, visit certain governments and countries whose first history time has assigned almost to *archæology*, note the advancement of science in each, the perfect order, system, and apparent perfection which long centuries have slowly and carefully instituted, beside being substantially aided and maintained by lavish appropriations from governmental treasuries. To glance back over the waters to his own home and country, reposing in its own unostentatious and unpretending position, while his vision is beclouded and obscured by the tinsel investments and vain show of royal trappings, and then to institute unfair comparisons and draw most unjust and unfavorable conclusions in relation to its scientific and other interests, are the natural results of a weak and surface mind. To forget that in all departments of our country the utilitarian principles and features are strongly engrafted upon, and interwoven with, our scientific, intellectual and civil systems, is another evidence of his want of capacity to understand our national proclivities, peculiarities and excellencies, and his utter ignorance of the condition of the different departments of his own land and country.

Medical science here has rested for its success and advancement almost entirely upon the individual efforts of its own disciples and votaries. The self-reliance of the members of the profession upon their own mental resources has been and still is one of the great elements in its rapid strides. The same disadvantages under which it has labored would have consigned it to an oblivious obscurity with any other people on the globe. The sparseness of the population, in the early history of our government, was *not* favorable to the collection and the systematic classification of facts, and hence the difficulty for a long time of advancing the condition of medical science. And yet the stern requirements of humanity and necessity demanded the exercise of scrutiny and observation on the part of each individual, but the want of Medical Journals denied them an opportunity of placing these facts upon record. The materials were therefore disjointed, and no common structure could well be formed. It is true that times have changed with the unparalleled improvement and progress of the country, which has brought with it abundant means of observation, and we maintain that these opportunities have been well improved, although we may not have reached to an approximation in excellence as boasted of by those upon whom the smiles of royal favor have been bestowed and the resources of its treasury have been lavished. There is no aristocracy in science in this country save that which springs from worth and talent. Each one thinks, investigates and concludes for himself, particularly those who have devoted their time and talents to the work of inquiry and research. He proceeds with his work of investigation regardless of illiberal criticism or condemnation from any quarter. No titled dignitaries to sit in umpire upon the opinions of men, or crush the aspirations of the humble and the obscure. We have no royal favorites, whose weight in the scale of influence can chill the hopes of a zealous and efficient student, and whose word of condemnation is but *annihilation.* We have no "*Sirs*" or notable physicians to the Emperor, *the King*, *the Queen*, or some "*coof*" of a lord, well mated to some more vain than sensible "*lady*"; but each one is on an equal footing, so far as advantages go, and each one is permitted, nay, encouraged in the strife to excel. The progress of improvement does not depend upon the whims and caprices of a few whose minds are made weak and almost imbecile by the "empty flatterings" of royal partiality, as in the old monarchies, but the responsibilities rest as heavily upon one member as upon another, and the position of each is only determined by the number, and more particularly, by the character of his productions. He has no fear of royal arrogance, but is cordially and promptly awarded that distinction he merits. The members of the profession *en masse* are his peers, and he looks upon them as the impartial arbiters of the value of his labors.

Hence here, and there, and almost every where, over this extended country, are found pillars and props with equal capacity to advance the interests of medical science by the results of their observations and research, and by their masterly contributions. Populous cities and towns grew up upon the Atlantic border, and here the work commenced, and for a long period the labor was confined to, and rested upon, those who had selected this field as the theatre of their action, and the science of medicine as the theme of investigation and elucidation. But now, to use the language of a certain class of liberal politicians, we know no *north*, no *south*, no *east* nor *west*; because in all sections of our common country there are dense human settlements whose wants and requirements demand for their welfare the highest attainments, and equal acquirements with those of any other.

These thoughts were set in motion upon looking over the assemblage of our brethren at the recent meeting of the Association.—Although all the medical talent of the nation was not there, still, from every part of the Union, there was a good representation. With deep interest we surveyed the countenances of may distinguished men, whose labors and devotion to the best interests of American Medical Science made us to yearn toward them with a feeling of gratitude and affection which one brother should feel for another, mingled with deep-felt admiration for their many qualities of head and heart. And while we sat in their midst indulging in a quiet survey of each, we felt a pride that there were those present who would adorn the profession in any country, and that yet behind in their respective fields of labor, there were others whose briliant career had rendered them conspicuous at home and abroad.

We say we felt proud, but quickly succeeding to this, there was also a feeling of chagrin, that any one would prove fratricide enough to decry his own profession, or by instituting unjust comparisons, lower the standard of medicine in his own country.

Were we competent to the task, we would give a sketch of the prominent men composing that body, with a history of their services and labors; but we shall hope that the task will be undertaken by some one who will give us a biography of the lives of the prominent men of the profession of the country, of the past and present times. Such a work would prove interesting as well as profitable, and we trust that some one competent to the performance of such a work will undertake it.

P. S.—Since penning the above, we observe that the "New Jersey Medical Reporter," has commenced the work in a series of articles. This is commendable, and trust that this excellent periodical will prosecute it. It will give the engraved portraits of many or all of those whose biography is published.

